# Maternal-Fetal Conflict During Infection: Lessons From a Mouse Model of Placental Malaria

**DOI:** 10.3389/fmicb.2019.01126

**Published:** 2019-05-24

**Authors:** Yash Pandya, Carlos Penha-Gonçalves

**Affiliations:** Instituto Gulbenkian de Ciência, Oeiras, Portugal

**Keywords:** placenta, malaria, maternal-fetal conflict, toll-like receptors, pregnancy

## Abstract

Infections that reach the placenta via maternal blood can target the fetal-placental barrier and are associated with reduced birth weight, increased stillbirth, miscarriage and perinatal mortality. Malaria during pregnancy can lead to infection of the placental tissue and to adverse effects on the unborn child even if the parasite is successfully cleared, indicating that placental sufficiency is significantly compromised. Human samples and animal models of placental malaria have been used to unravel mechanisms contributing to this insufficiency and have implicated molecular pathways related to inflammation, innate immunity and nutrient transport. Remarkably, fetal TLR4 was found to take part in placental responses that protect the fetus, in contrast to maternal TLR4 responses that presumably preserve the mother‘s health but result in reduced fetal viability. We propose that this conflict of fetal and maternal responses is a determinant of the clinical outcomes of placental malaria and that fetally derived trophoblasts are on the front lines of this conflict.

## 1. Introduction

Pregnant women are at a higher risk of malaria infection (Espinosa et al., 2000; Lindsay et al., 2000). Infection by *Plasmodium falciparum* contributes to adverse outcomes including premature delivery, intra-uterine growth restriction, stillbirth and perinatal death alongside worsened maternal anemia and increased maternal parasite loads (Menendez et al., 2000; Crocker et al., 2004). These outcomes have been found to occur in pregnancies several months after clearance of the parasite, illustrating that treatment of the infection alone may be of little benefit (Schmiegelow et al., 2017).

Placental infection is a key determinant of these outcomes and the molecular basis of placental malaria pathology has been intensively studied (recently reviewed by Fried and Duffy, 2017). Parasite sequestration in the placenta is the primary pathological event, and, in the case of *Plasmodium falciparum*, is primarily mediated by infected erythrocytes binding to chondroitin sulfate A on the surface of syncytiotrophoblasts (Fried and Duffy, 1996; Abrams et al., 2003; Miller et al., 2013; Moya-Alvarez et al., 2014). Interactions between infected erythrocytes and placental tissue trigger significant infiltration of maternal inflammatory cells (Fried and Duffy, 2017) and alterations in the profile of cytokines secreted in the placenta, namely increases in TNFα and IFN-γ which are linked to adverse pregnancy outcomes (Moormann et al., 1999; Muehlenbachs et al., 2007).

Placentas from infected women show functional alterations including reduction in the activity of system A, a group of sodium dependent amino acid transporters which actively uptake small amino acids into the trophoblast layer (Boeuf et al., 2013). Placental glucose transporter activity is reduced when infection is accompanied by intervillositis (Chandrasiri et al., 2013). Malaria also reduces placental megalin, a transporter for a vast array of proteins (Lybbert et al., 2016). These nutrient transport pathways depend on an adequate placental blood supply to function effectively and placentas from women infected with malaria exhibit reduced placental perfusion (Dorman et al., 2002; Brabin and Johnson, 2005), impaired trophoblast invasion (Umbers et al., 2013), and alterations in various angiogenic factors within the placenta (Ataíde et al., 2015) which corroborates suboptimal placental perfusion.

More recently, a prospective study revealed that blood levels of L-arginine, a precursor to the potent vasodilator nitric oxide, were reduced in women with placental malaria while levels of dimethylarginine, an inhibitor of nitric oxide biosynthesis, were increased. These changes were strongly correlated with worse birth outcomes (McDonald et al., 2018). Earlier studies have highlighted a potential role for vascular endothelial growth factor (VEGF) and its receptors in the response to placental malaria in primigravid mothers, with soluble receptors for VEGF being more abundantly expressed in the placenta. This further implicates circulatory impairments in the disease pathology and provides the first evidence that placental responses to infection may not be in harmony with maternal responses, as maternal cells in the placenta showed elevated VEGF levels whereas fetal syncytiotrophoblasts produced more sVEGFR1, reducing VEGF bioavailability (Muehlenbachs et al., 2006).

These findings suggest that the intertwining of inflammatory signals, vasoregulatory systems, and nutrient transport pathways in the placenta are critical components of human placental malaria pathophysiology. However, experimental demonstration of the pathogenic mechanisms operating in the placenta relies on available mouse models of disease. In this perspective article we explore evidence generated from a mouse model of acute placental malaria that highlights the role of toll-like receptor 4 (TLR4) in controlling the outcomes of pregnancy ergo providing an interesting example of infection provoking conflict between the mother and the unborn child.

## 2. Pathogenesis of Murine Acute Placental Malaria

Several murine experimental systems have been used to model specific aspects of malaria in pregnancy, but extrapolations to human disease should be considered with caution (Hviid et al., 2010). These experimental systems model different aspects of malaria in pregnancy, including: use of recrudescent *Plasmodium berghei* ANKA to study maternal susceptibility to infection (Marinho et al., 2009); a system using *P. berghei* K173 strains to infect mice both prior to and during gestation as would occur in high transmission settings (Van Zon and Eling, 1980); and a system making use of *Plasmodium chabaudi* which allows for the study of infections in early stages of pregnancy (Poovassery et al., 2009). Here, we will focus on a model which makes use of *Plasmodium berghei* infection during gestation and which models acute malaria during pregnancy in women. Briefly, infecting naïve, primigravid BALB/c females with 10^6^ *P. berghei ANKA* infected erythrocytes intra-venously on the 13th day of gestation results in severe disease outcomes, such as intra-uterine growth restriction, decreased fetal viability, post-natal growth impairment and increased maternal parasitemia and anemia (Neres et al., 2008). Similar results are obtained with the use of the NK65, K173 and ANKApm4 lines of *P. berghei* in primigravid C57BL/6 mice, following the same mating and dosage protocols (Rodrigues-Duarte et al., 2012). Examination of the placentas with acute infection revealed an accumulation of infected erythrocytes and hemozoin in the blood sinusoids (Sharma et al., 2012b), thickening of the labyrinthine zone, deposits of hemozoin, fibrinoid necrosis, hyperplasia of the syncytiotrophoblasts, reduced blood sinusoid area, and a significant infiltration of maternal macrophages and monocytes (Neres et al., 2008).

This model has allowed investigation of the underpinnings of placental dysfunction, particularly by linking inflammatory responses to alterations in angiogenic and vasoregulatory pathways. This is illustrated by descriptions of increases in the amounts of angiopoietin 1 and in the ratio of angiopoietin 1 to angiopoietin 2 in infected placentas belonging to viable, low birth weight offspring (Silver et al., 2010) as well as by the reduced expression of bradykinin receptor B2 and NOS3 genes, both known to be involved in vasodilatory responses (de Moraes et al., 2018). Human studies have also revealed that infection with *P. falciparum* during pregnancy increases levels of these angiopoietins and complement C5a while reducing nitric oxide bioavailability (Conroy et al., 2013; McDonald et al., 2018). Genetically ablating C5a receptor in mice infected with *P. berghei* during pregnancy increased placental vascular branching and ameliorated the increase in resistance to flow caused by infection (Conroy et al., 2013). Similar results were obtained by dietary supplementation with L-arginine, a nitric oxide precursor (McDonald et al., 2018). Furthermore, intra-vital imaging (Lima et al., 2014) has revealed how infected erythrocytes accumulate in areas of slower flow in the placental labyrinth, possibly adhering to, or being phagocytosed by, the syncytiotrophoblasts and suggesting that infection impairs local circulatory regulation (de Moraes et al., 2013). Additionally, oxidative stress has been implicated in malaria during pregnancy, with a combination of increased lipid perodixation (Sharma et al., 2012a), decreased catalase activity and increases in apoptosis markers being observed in the placentas of infected mice while the absence of alterations in Fas expression and Caspase 8 indicate that the damage caused is primarily via the mitochondrial pathway of apoptosis (Sharma et al., 2012b). Treating the infected mice with chloroquine or with sulfadoxine pyrimethamine abrogated oxidative stress, apoptosis, and placental damage, consequently improving birth weight. Interestingly, anti-malarial treatment of mice with placental malaria did not improve fetal survival, indicating that placental insufficiency is not recoverable by parasite clearance alone (Sharma and Shukla, 2014). This raises the possibility that innate immune stimulation during pregnancy results in enduring placental dysfunction. Here, we argue that responses mediated by Toll-like receptors (TLRs), particularly TLR4, have a decisive impact on the development of placental pathologies during infection.

## 3. TLR4, Malaria, and Pregnancy

**Figure 1:**
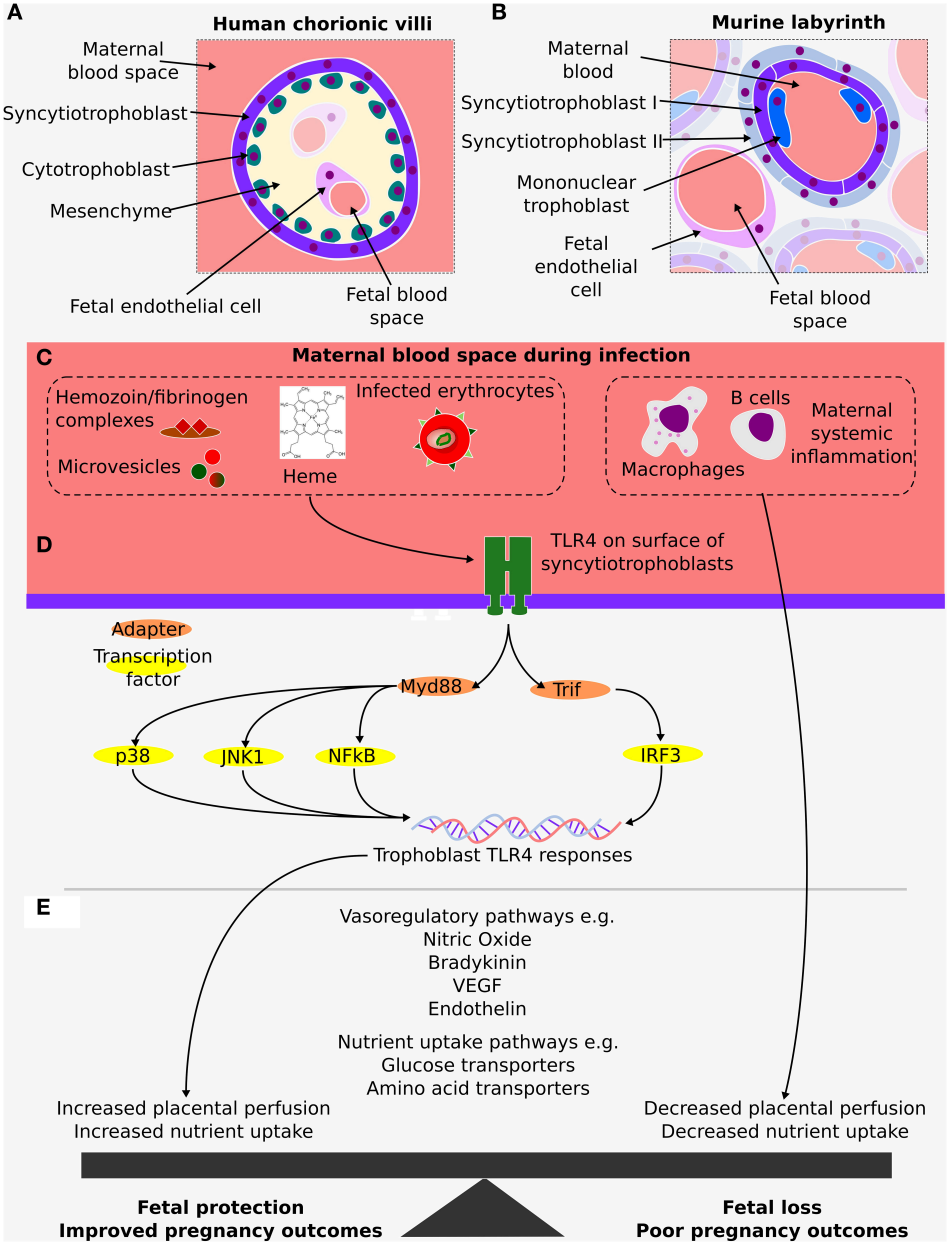
**(A)** Cross section of a human placental terminal chorionic villus, which is part of highly branched tree of fetally derived tissue anchored in the chorionic plate and surrounded by maternal blood. At the terminal chorionic villi, the placental barrier is composed by the endothelial cells enclosed in mesenchyme followed by a layer of cytotrophoblasts and an outermost layer of syncytiotrophoblasts in contact with maternal blood. **(B)** Cross section of the analogous murine tissue, the placental labyrinth where the maternal fetal barrier is made up of a layer of fetal capillary endothelial cells, two layers of syncytiotrophoblasts and a discontinuous layer of mononuclear trophoblasts that is in direct contact with maternal blood. **(C)** In both the human and murine placentas, fetally derived syncytiotrophoblasts come into direct contact with maternal blood, an important similarity, as during a malaria infection, these are the fetal cells exposed to maternal inflammatory mediators and to components of parasite origin such as infected erythrocytes and microvesicles. **(D)** TLR4 detects and responds to several of these stimuli via either MyD88 or TRIF, activating several transcriptional factors including p38 and IRF3. **(E)** Transcriptional changes in the syncytiotrophoblast contribute to alterations in local production of vasoactivators such as nitric oxide, bradykinin and endothelin as well as altering nutrient transport pathways in a manner which result in fetal protection. In contrast, maternal responses impair these pathways and worsen the outcomes of pregnancy.

TLRs are a class of pattern recognition receptors involved in the detection of, and in the response to, pathogen and damage associated molecular patterns (PAMPs and DAMPs) by activation of downstream signaling pathways which induce immunity mediators including pro-inflammatory cytokines and interferons. TLR signaling makes use of either of two adaptor proteins, MyD88 or TRIF, with TLR4 being unique in its ability to use both of these pathways (Figure 1D) (Lu et al., 2008; Kawasaki and Kawai, 2014).

TLRs play a significant role in the response to malaria infection, participating in the recognition of glycosylphosphatidylinositol anchors, peroxiredoxin and fibrinogen/hemozoin complexes as well as host derived microvesicles and heme (Eriksson et al., 2014; Gazzinelli et al., 2014) (Figure 1C). TLR4 polymorphisms have also been associated with disease severity, particularly the hyporesponsive polymorphisms *Tlr4* Asp299Gly and *Tlr4* Thr399Ile which predispose children to severe malaria (Schmitt et al., 2002; Mockenhaupt et al., 2006a). However, contrasting results suggested that these polymorphisms may be beneficial in adults (Esposito et al., 2012; Basu et al., 2014) and a recent meta-analysis found no association between *Tlr4* Asp299Gly and the outcomes of malaria (Dhangadamajhi et al., 2017). In mice, TLR4 has been implicated in dendritic and mast cell activation during malaria (Furuta et al., 2008; Seixas et al., 2009), potentially contributing to the resistance of DBA/2 mice to infection with *P. yoelii*, although TLR4 has not been linked to the pathology of experimental cerebral malaria (Togbe et al., 2007).

### 3.1. TLR4 in Pregnancy

Several TLRs are expressed in fetally derived placental tissues and surrounding maternal tissue Koga and Mor (2010) but cumulative evidence suggests a specific role for TLR4 in the outcomes of pregnancy. It has been observed that fetoplacental TLR4 expression is decreased in miscarriages (Kolben et al., 2019) and in preeclampsia patients (Kulikova et al., 2016), while the *Tlr4* Asp299Gly polymorphism in the fetus is associated with severe prematurity (Rey et al., 2008). In contrast, increases in TLR4 expression on maternal monocytes, which may be responding to fibrinogen, are correlated with spontaneous preterm labor (Pawelczyk et al., 2010; Al-ofi et al., 2014), and increased expression in maternal decidua has been linked to recurrent miscarriages (Li et al., 2016). These data suggest that the role of maternal and fetal TLR4 in pregnancy may be in opposition, with reductions in fetal activity and/or increases in maternal activity being detrimental to the outcomes of the pregnancy.

Various mouse models have illustrated the role of TLR4 in pregnancy associated infections and disorders, including malaria (Barboza et al., 2017; Rodrigues-Duarte et al., 2018), bacterial infections (Liu et al., 2007; Arce et al., 2012; Chin et al., 2016), lipopolysaccharide exposure (Breen et al., 2012; Wahid et al., 2015) and uterine ischemia (Thaete et al., 2013). It should be noted that these studies (barring that by Rodrigues-Duarte et al., 2018) have focused on completely eliminating TLR4 signaling and, consequently, do not differentiate between fetal and maternal TLR4 responses. Furthermore, increased fetal TLR4 activity has been found in models of maternal ethanol-induced inflammation (Zheng et al., 2014) as well as in maternal cigarette smoke exposure (Chan et al., 2016). These studies support that maternal factors may be contributing to alterations in fetal innate immune responses as well as having direct impacts on the outcomes of pregnancy. Still, in all of these cases, the downstream actions of TLR4 are yet to be fully understood.

### 3.2. TLR4 in Placental Malaria

The role of TLR4 in malaria during pregnancy has also been examined in genetic association studies. The *Tlr4* Asp299Gly and *Tlr4* Thr399Ile maternal polymorphisms appeared more frequently in women who had a higher parasitemia and severe anemia, and translated to a significantly increased risk of low birth weight. They had no impact on prematurity, viability or the incidence of placental malaria (Mockenhaupt et al., 2006b), suggesting that maternal TLR4 takes part in responding to infection, but may not be linked to severe placental dysfunction during malaria. In mice, examination of TLR4 was preceded by work on MyD88, which was shown to contribute to reductions in placental vascular space and fetal weight (Barboza et al., 2014). Genetic ablation of several TLRs which use this adaptor protein demonstrated that alterations in vascular space, TNFα production and detrimental outcomes are directly linked to TLR4 (Barboza et al., 2017).

Genetic ablation of TLR4 confers striking protection from fetal death induced in murine placental malaria. The roles played by fetally derived placental cells in protecting fetal viability were discerned by comparing pregnancy outcomes when the fetal placenta either expressed TLR4 or did not. As expected, we observed improvements in fetal viability in TLR4KO females which were carrying TLR4KO offspring, suggesting that the TLR4 response to infection was deleterious to the fetuses, as observed in the other mouse models of disease during pregnancy mentioned above. Unexpectedly, TLR4KO females carrying placentas expressing fetally derived TLR4 showed further improvements in the outcomes of pregnancy with stillbirth rates similar to those of uninfected mothers (Rodrigues-Duarte et al., 2018). This showed that TLR4 in the fetal compartment was protective for the litter, whereas having it in the maternal compartment was harmful, a conflict which is yet to be investigated in other infections. Although the mechanism behind this protection has not been fully elucidated, alterations in glucose (Chandrasiri et al., 2013) and amino acid (Boeuf et al., 2013) transport, observed using human samples from malaria infected individuals, suggest that a conflict may arise over the allocation of metabolic resources. On the other hand, altered nitric oxide bioavailability (McDonald et al., 2018) and VEGF levels further point toward a role for fetal TLR4 responses in regulating placental perfusion.

While other models of infection during pregnancy have not yet been interrogated in a manner which allows for the disentanglement of fetal and maternal responses, there are strong similarities between the impact of malaria infection on placental TLR4 and the impact of a variety of other pathogens. In wild type mice infected with *P. berghei*, the amount of TLR4 protein detected in the placenta is significantly increased (Barboza et al., 2017), a pattern which is replicated with *Campylobacter rectus, Porphyromonas gingivalis* (Arce et al., 2009) and murine cytomegalovirus infections (Liao et al., 2018). It would be particularly important to determine if the maternal component is responsible for the outcomes of these infections and assess the impact of maintaining the fetal response. Bearing in mind that the first point of contact for fetally derived TLR4 with infectious agents in the maternal blood is at the placental barrier, examining fetally derived placental cells may be key to better understand the conflict with maternal responses.

## 4. Primary Trophoblast Responses to Infection

The fetal cell type directly in contact with maternal blood in both humans and mice are the syncytiotrophoblasts. They are responsible for the exchange of nutrients and waste, as well as forming the barrier between maternal and fetal circulation (Zeldovich et al., 2013; Blackburn, 2015; Maltepe and Fisher, 2015) (Figures 1A,B). While TLRs are found in fetally derived cells in the placenta (Koga and Mor, 2010), their expression and activity is greatly altered in placental cell lines, showing a lack of suitability for examining certain aspects of trophoblast behavior (Amirchaghmaghi et al., 2013; Gierman et al., 2015), and highlighting a requirement for the employment of primary cultures.

Although they are rarely used for studies with malaria (Lucchi et al., 2006, 2008), primary human trophoblasts have been successfully isolated and used to examine responses to a variety of infectious agents such as *Brucella spp*., zika virus and *Toxoplasma gondii* (Salcedo et al., 2013; Aagaard et al., 2017; Ander et al., 2018), the latter being further examined in villous explants (Ander et al., 2018). This experimental system has also been used to study *Trypanosoma cruzi* infection (Díaz-Luján et al., 2016; Medina et al., 2018; Triquell et al., 2018), which is known to provoke changes in several immune related genes during pregnancy (Juiz et al., 2018). Trophoblasts are reported to show changes in amino acid and glucose uptake in response to lipopolysaccharide (a TLR4 ligand) (Liong and Lappas, 2017), pathways which are also altered in human placental malaria samples (Boeuf et al., 2013; Chandrasiri et al., 2013). Therefore, the use of primary human trophoblasts to study responses to malaria infection may provide insights into the pathology of placental malaria.

As an alternative, murine trophoblasts, isolated from term placentas or of stem cell origin, represent a powerful tool for studying infection during pregnancy. TLR4 responses have been examined in cell culture studies using *P. berghei*. These studies have revealed a reduction in the amount of trophoblast-associated parasite in absence of TLR4 as well as marked reduction in the expression of *Ifnar1* in these cells (Rodrigues-Duarte et al., 2018). Along the same lines, *Listeria monocytogenes* has been shown to be taken up by trophoblast giant cells in a MAPK dependent manner, using innate sensing systems heavily influenced by TLR2, subsequently downregulating HO-1 and resulting in cell death (Hashino et al., 2015). In a study not linked to TLRs, trophoblasts exposed to *Toxoplasma gondii* have been demonstrated to undergo apoptosis as well as alter production of various cytokines, with increased oxidative stress and subsequent mitochondrial damage (Liu et al., 2013; Xu et al., 2015). Thus, it is clear that trophoblasts respond to pathogens infecting maternal blood, raising the interesting possibility that they are the initiators of fetal protective responses in placental infections, particularly when the pathogens do not cross the placental barrier.

## 5. Concluding Remarks

It is expected that a variety of pathways are impacted upon during placental infection, affecting inflammatory responses, nutrient transport and vasoregulatory responses, and contributing to placental insufficiency that leads to poor pregnancy outcomes. A key finding from the acute murine placental malaria model has been the importance of TLR4 in the determination of the outcomes of pregnancy. Interestingly, this trait is shared with several other models of disease in pregnancy that also show signs of placental dysfunction. The strong pathological similarities between these models supports the proposal that innate immune recognition by the placental tissue may improve fetal survival in other infections.

Taken together, the work highlighted here leads us to propose that maternally driven TLR4 responses to malaria infections, and other illnesses during pregnancy, are deleterious for the fetus, impairing nutrient/waste exchange, hampering placental perfusion and worsening the outcomes of pregnancy. In contrast, feto-placental TLR4 responses are protective, as demonstrated by Rodrigues-Duarte et al. (2018) and may compensate for the maternal actions by activating mechanisms to increase nutrient uptake and placental perfusion (Figure 1E). This re-inforces the notion that malaria infection induces maternal-fetal conflict, as proposed by Muehlenbachs et al. (2006), in their examination of soluble VEGF receptor 1 in human placental malaria, but further hints that fetal responses which preserve placental function are initiated by innate immune recognition and downstream signaling to effector mediators in trophoblasts.

The syncytiotrophoblasts are at the front line of this conflict between mother and fetus and their examination, using *in vitro* and *in vivo* experimental systems, as well as in human tissue samples, will be required for the identification of critical components of the fetal protective responses. Making use of the vast range of existing genetic mouse models in conjunction with the gamut of infection systems which have been developed for the study of pathological pregnancies, will provide a clearer understanding of the mechanisms that protect from adverse outcomes of pregnancy.

## Author Contributions

YP contributed to writing the paper. CP-G contributed to writing the paper.

### Conflict of Interest Statement

The authors declare that the research was conducted in the absence of any commercial or financial relationships that could be construed as a potential conflict of interest.
